# Visual Sorting of Express Packages Based on the Multi-Dimensional Fusion Method under Complex Logistics Sorting

**DOI:** 10.3390/e25020298

**Published:** 2023-02-05

**Authors:** Chuanxiang Ren, Haowei Ji, Xiang Liu, Juan Teng, Hui Xu

**Affiliations:** 1 College of Transportation, Shandong University of Science and Technology, Qingdao 266590, China; 2 School of Automobile, Tongji University, Shanghai 201804, China

**Keywords:** object detection, Mask R-CNN, point cloud, multi-dimension fusion, express package, logistics sorting

## Abstract

Visual sorting of express packages is faced with many problems such as the various types, complex status, and the changeable detection environment, resulting in low sorting efficiency. In order to improve the sorting efficiency of packages under complex logistics sorting, a multi-dimensional fusion method (MDFM) for visual sorting in actual complex scenes is proposed. In MDFM, the Mask R-CNN is designed and applied to detect and recognize different kinds of express packages in complex scenes. Combined with the boundary information of 2D instance segmentation from Mask R-CNN, the 3D point cloud data of grasping surface is accurately filtered and fitted to determining the optimal grasping position and sorting vector. The images of box, bag, and envelope, which are the most common types of express packages in logistics transportation, are collected and the dataset is made. The experiments with Mask R-CNN and robot sorting were carried out. The results show that Mask R-CNN achieves better results in object detection and instance segmentation on the express packages, and the robot sorting success rate by the MDFM reaches 97.2%, improving 2.9, 7.5, and 8.0 percentage points, respectively, compared to baseline methods. The MDFM is suitable for complex and diverse actual logistics sorting scenes, and improves the efficiency of logistics sorting, which has great application value.

## 1. Introduction

At present, there are many disorderly stacked and diverse types of express packages in logistics transportation, forming a complex logistics sorting background. As a result, it is difficult to improve the sorting efficiency of express packages in this scene, which greatly affects the progress of logistics transportation. With the rapid development of deep learning theory, object detection and recognition technology based on machine vision has been gradually applied to the logistics industry [1,2], but there are still deficiencies in the detection and grasping of express packages under complex logistics sorting, which makes the sorting one of the main weaknesses in the development of the logistics industry at the present stage.

Automatic sorting of express packages [3] has been a mainstream solution in logistics transportation, where object detection technology is applied to obtaining information such as position, category, segmentation mask [4] and posture [5], and then the intelligent sorting robot will grab packages accurately by locating and tracing them [6,7]. Traditional object detection technologies [8] such as key point detection, Histogram of Gradient [9] and Scale-Invariant Feature Transform [10] are not suitable for detecting in complex scenes like shadows [11,12] or on blurred images [13] due to poor generalization and slow execution speed. While object detection algorithms based on Convolutional Neural Network (CNN) have more detection accuracy and are gradually applied in practice [14,15], they have been divided into the two-stage algorithm and the one-stage algorithm. The two-stage algorithms such as Region-CNN (R-CNN) [16], Fast R-CNN [17], Faster R-CNN [18] and Mask R-CNN [19] have better detection accuracy but take longer inference time and lack in real-time detection compared to one-stage algorithms represented by SSD (Single Shot Multibox Detector) [20], YOLO (You Only Look Once) [21] and RetinaNet [22]. Compared with the traditional ones, object detection algorithms based on CNN can extract the features of the target more effectively, and adapt to detection tasks in specific scenes like small target detection [23,24], obscured target detection [25] and multi-target detection [26].

In terms of logistics transportation, early studies mainly focused on the application of object detection and recognition. For example, Hwang et al. [27] applied the object detection algorithm to the recognition of goods to realize the automatic loading of trucks, and Gou et al. [28] studied the dataset image synthesis method of cargo to load and unload cartons based on deep learning, so as to improve the target recognition. The objects in the above research were cartons with regular appearances, which are detected and recognized more easily than multiple types of express packages in the complex logistics sorting scene. In terms of visual sorting, Zuo et al. [29] studied the location detection of targets in a scene stacked with objects by combining machine vision and a deep learning algorithm and then controlled the sorting robot to grasp it. Han et al. [30] proposed a visual sorting method based on multi-modal information fusion to improve object detection and grasp accuracy of the manipulator. Both of these approaches aim to solve the problem of object grasping in complex scenes, but there are not only the problems of single type and regular shape of the target, but also the simple and ideal experimental background, which is greatly different from a real complex sorting scene. In terms of the determination of optimal grasping position of the target, Han et al. [31,32] proposed a robot sorting method based on a deep neural network where the geometric center had been calculated from four key points determining the final grasping position. However, this method has limitations for objects with irregular shape and uneven surface. What’s more, as the actual logistics sorting scene is quite complex and changeable, the methods above find it difficult to satisfy the requirements of a real situation where express package sorting operates.

In the actual logistics transportation sorting scene, the background of express package detection and recognition is more complex and restricted by various factors, which can be mainly divided into external environment factors and express package itself factors. The ambient light is one of the external environment factors that influences detection effects of the target [33,34,35], for example, strong light makes the package surface reflect and be overexposed, while uneven lighting conditions lead to a large range of shadows. These external influences make the texture features of the package surface fade, fuzz, disappear or be confused with the background, thus affecting the detection and recognition of the target, reducing the detection accuracy and image segmentation quality. Poor lighting conditions will also affect the RGB-D camera’s extraction of target depth information, and then affect the generation and transformation of 3D point cloud data [36,37,38]. In addition to external environment factors, the target to be detected also has a great impact on object detection and instance segmentation [39,40,41]. In actual logistics transfer center scenes, there are a large number of packages with different shapes, colors and materials stacked in a disorderly manner. Some of them are similar in appearance, such as shape and color, which are difficult to distinguish, or are composed of the same material being overlapped or obscured. Moreover, some packages are prone to reflect lights, imaging unclearly and appearing seriously deformed due to special materials, which are difficult to identify. Furthermore, these packages usually appear in dense distributions, unevenly or dispersedly. In general, under the combined influence of these two adverse factors, a complex logistics sorting background has been formed, which is quite different from those of previous studies.

Although effective methods had been proposed to solve corresponding problems in the studies above, the influences of various targets and backgrounds of research were ignored, leading to disadvantages in detection and sorting under complex logistics sorting. In order to improve the sorting efficiency of express packages, a multi-dimensional fusion method for visual sorting is proposed that is suitable for diverse types of packages in complex logistics sorting scenes. Mask R-CNN is applied to the 2D detection task, from which the segmentation mask is combined with 3D point clouds to determine the sorting vector and the optimal grasping position of the express package in real time. Lastly, an experiment on robot sorting is carried out to verify the progress of the proposed method on sorting efficiency. It is hoped that the MDFM can improve the efficiency of logistics sorting and promote the development of the logistics industry.

## 2. Method

**Figure 1:**
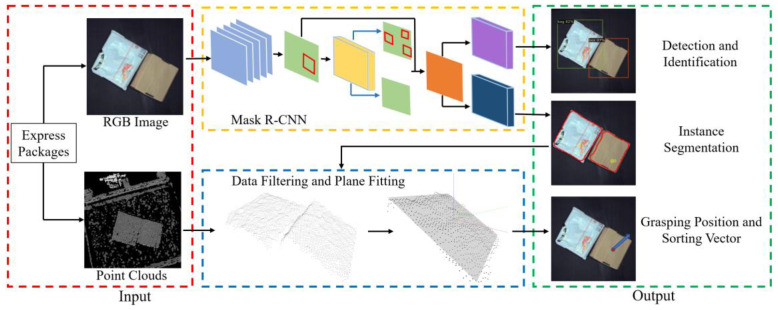
Overall framework of MDFM.

Due to the uneven surface of most express packages, especially those easily deformed packages such as bags, and the complex situation of disordered stacking and overlap, the traditional method that estimates the pose and determines the grasping position of packages based on point cloud is difficult to apply to the complex logistics sorting scene. To this end, the multi-dimensional fusion method is proposed, in which Mask R-CNN is adopted and 3D point cloud data is used, and its overall framework is shown in Figure 1.

In MDFM, Mask R-CNN is designed and applied to detect and recognize different kinds of express packages, obtaining information of the category and instance segmentation. Point cloud data filtering is designed to acquire accurate point clouds of the package grasping surface, which combines the boundary information of 2D instance segmentation generated form Mask R-CNN to accurately filter the 3D point clouds of the package grasping surface. It can reduce the interference of non-grasping surfaces and the other packages on the point cloud extraction. Then, the ordinary least squares method is used to conveniently and quickly fit the point clouds into a virtual plane, and the normal vector of the plane is obtained to determine the sorting vector of the package. At last, the geometric center of the original surface is mapped to the fitting plane, where the final optimal grasping position is located.

### 2.1. Detection on Express Packages

Mask R-CNN is applied to the 2D detection task in MDFM considering its multifunctional ability in detection and adaptability to complex scenes, through which category classification, bounding box regression and instance segmentation can be carried out, possessing the practicability for the detection task in complex backgrounds.

Affected by the complexity of the actual logistics sorting scene, the accuracy of the one-stage object detection algorithm is lower than that of the two-stage object detection algorithm. Compared with other two-stage target detection algorithms, Mask R-CNN can detect and recognize targets and segment instances more precisely at the same time, dividing individual package units accurately, which is more conducive to the automatic sorting of express packages.

### 2.2. Method for Point Cloud Data Filtering

After using the Mask R-CNN to accurately process the express packages, information such as the type and quantity of packages to be sorted and the boundary of the segmentation mask can be obtained at the 2D level. Combined with the 3D information such as the coordinate position, pose and grasping position of the package, the sorting robot can be applied for accurate and fast automatic operation.

Data filtering refers to the targeted filtering of 3D point clouds collected by the RGB-D camera, which is generally divided into two parts: the point cloud filtration of all express packages to be sorted, and the point cloud filtration of each express package grasping surface. Due to the impact of complex logistics sorting backgrounds, point clouds of express packages collected by the RGB-D camera often contain other interference factors, such as the conveyor belt, sorting table or even irrelevant packages outside the sorting range. By setting a range threshold of filtration, the point clouds of other objects outside the detection and sorting range are eliminated, and only the point clouds of express packages to be sorted will be retained, which also prepares for the next step of combining boundary information to filter point clouds of the grasping surface.

**Figure 2:**
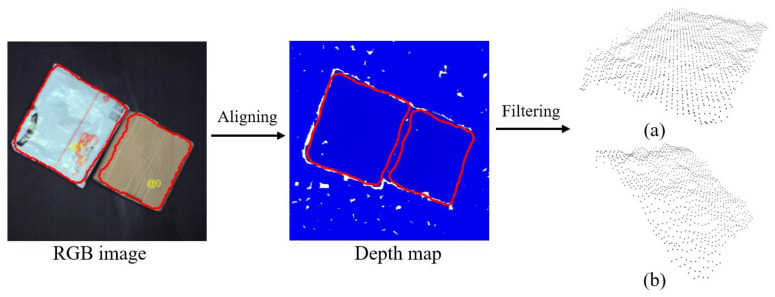
Schematic diagram of point cloud data filtering on the grasping surface. (**a**) Point clouds of bag (**b**) Point clouds of box.

As shown in Figure 2, the segmentation mask of the grasping surface can be accurately generated on a single express package through the instance segmentation of Mask R-CNN, and the boundary contour of the mask can be drawn on the RGB image. Next, the RGB image is aligned with the depth image, and the boundary contour is called to divide the range of the grasping surface of the package to be sorted on the depth map, and then this part of depth information will be converted into the corresponding point clouds. After this, the accurate filtration of the grasping surface has been realized, which also reflects the combination of visual information and 3D information. In addition, calibration of the RGB-D camera is required before the detection task to avoid influences caused by camera distortion.

### 2.3. Plane Fitting and Sorting Information

In order to improve the efficiency of the logistics sorting, it is necessary to provide accurate sorting information like the grasping position and sorting vector of packages, which means plane fitting ought to be carried out based on the point clouds that are filtered from the object grasping surface, and then the position of the plane center and the normal vector will be calculated. Suppose the set of the grasping surface points is *P*, then the set *P* can be represented by Formula (1).

(1) $$\begin{array}{c} P=\left\{\left(x_{i},y_{i},z_{i}\right),\;i=0,\;1,\;2,\ldots ,n-1\right\}\; \end{array}$$

where (x,y,z) represents the coordinate of the point cloud in the set *P*, $\left(x_{i},y_{i},z_{i}\right)$ represents the coordinate of the *i*th point cloud in the set *P*, *n* represents the number of point clouds composing the grasping surface.

Given that the points in the set *P* represent *n* discrete points in the grasping surface, the ordinary least squares method is used to fit them into a new plane. The calculations in detail are shown as follows.

The expression of the ordinary plane can be expressed as:

(2) $$\begin{array}{c} aX+bY+cZ+d=0 \end{array}$$

where *X*, *Y*, and *Z* represent the values of the *x*, *y*, and *z* axes, respectively, at any point on the plane, *a*, *b*, *c*, and *d* represent arbitrary constants.

Supposing that c≠0, making $A=-\frac{a}{c}$, $B=-\frac{b}{c}$, $C=-\frac{d}{c}$, the new fitting plane expression can be expressed as:

(3) $$\begin{array}{c} AX+BY+C=Z \end{array}$$

If there are *m* (*m* ≤ *n*) points in set *P*, according to the principle of the ordinary least squares method, making the quadratic sum of *z*-axis errors between points and corresponding points on the fitting plane minimize, as shown in Formula (4).

(4) $$\begin{array}{c} \left(A,B,\;C\right)=argmin\overset{m-1}{\underset{i=0}{\sum }}{\left[Z\left(A,\;B,\;C\right)-z_{i}\right]}^{2} \end{array}$$

where *m* represents the number of point clouds that satisfy Equation (4), $z_{i}$ represents *z*-axis value of the *i*th point cloud.

After solving the ordinary least squares problem expressed by Formula (4), unknown quantities *A*, *B* and *C* can be worked out. The expression of the fitting plane can be obtained, and the normal vector of the fitting plane is:

(5) $$\begin{array}{c} \vec{v}=\left(A,B,-1\right) \end{array}$$

After obtaining the expression of the fitting plane expression, the optimal grasping position of the plane can be calculated and determined, as shown in the following formulas:

(6) $$\begin{array}{c} \left(x^{\prime },y^{\prime },z^{\prime }\;\right)=\left(\frac{\sum_{i=0}^{n-1}x}{n},\frac{\sum_{i=0}^{n-1}y}{n},\frac{\sum_{i=0}^{n-1}z}{n}\right) \end{array}$$

(7) $$\begin{array}{c} \left(X^{\prime },Y^{\prime },Z^{\prime }\;\right)=\left(x^{\prime },y^{\prime },Ax^{\prime }+By^{\prime }+C\right) \end{array}$$

where $\left(x^{\prime },y^{\prime },z^{\prime }\;\right)$ represents the coordinate of the point which is the geometric center of the original surface, $\left(X^{\prime },Y^{\prime },Z^{\prime }\;\right)$ represents the coordinate of the point on the fitting plane mapped from $\left(x^{\prime },y^{\prime },z^{\prime }\;\right)$ by Expression (3), namely the optimal grasping position of this plane.

**Figure 3:**
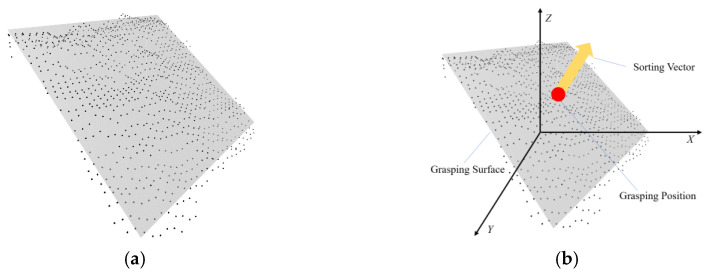
Diagram of fitting plane and sorting information. (**a**) Plane fitting. (**b**) Sorting information.

The 3D point clouds of the grasping surface are accurately filtered through the boundary information of 2D instance segmentation, and the virtual plane is quickly fitted to determine the sorting vector and the optimal grasping position, as shown in Figure 3. The processing of plane fitting is not only beneficial for improving the grasping accuracy of express packages in a disordered distribution, but also to a certain extent reduces the impact of adverse lighting conditions in complex scenes, resulting in the absence of point clouds on the package surface, and finally improves the overall sorting efficiency.

## 3. Experiment and Analysis

### 3.1. Experiment on Mask R-CNN

In MDFM, the detection accuracy of identification and instance segmentation has great influence in sorting strategies and point cloud data filtering. Therefore, evaluation of Mask R-CNN is necessary to verify its detection capability in complex scenes.

#### 3.1.1. Data Processing and Dataset

**Figure 4:**
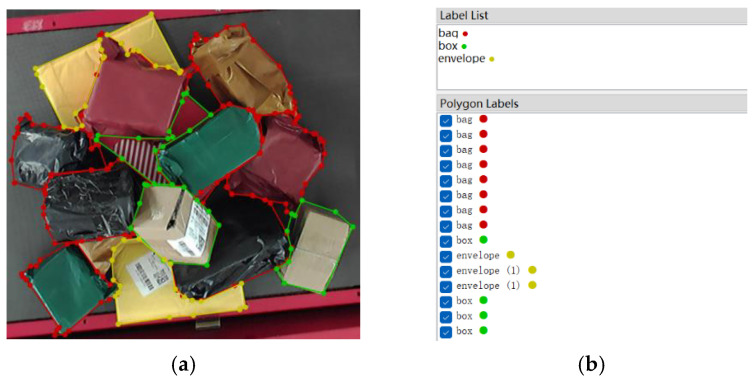
Diagram of labeling dataset images. (**a**) Labeling of different targets. (**b**) Detailed information of labeling.

The dataset images are obtained from the RGB-D camera located above the sorting table during the sorting of express packages under actual complex logistics sorting. The images include the three most common types of express packages in logistics transportation, namely box, bag and envelope. Labelme, a software used for labeling, is used to make the dataset. In each image, a single target is distinguished from the background by the polyline along the edge of itself, and the category label is added to the target at the same time. The same type of the express package corresponds to the same label, which can be displayed visually by the color of the polylines, as shown in Figure 4a. After the labeling of an image is completed, the software will display the type of labels and the corresponding number, as shown in Figure 4b.

In the experiment, 700 images are screened out and labeled to make the dataset, containing all kinds of express packages with different sizes, colors, shapes, and surface texture features. In the dataset, images of express packages in several complex sorting scenes are included, and different light conditions and stack status of packages are considered. The dataset is divided into the training set and the validation set with the ratio of 6:1, and the quantity distribution of these three categories of targets is counted, as shown in Table 1.

#### 3.1.2. Experiment Environment and Evaluation Indexes

In this experiment, Python3.8.5 programming was used and the detectron2 object detection framework was established based on Pytorch1.7.1 and CUDA11.0 version environment. The training, validating and testing of Mask R-CNN are carried out on NVIDIA GeForce RTX-3060 GPU.

Average precision (AP) and mean average precision (mAP) are used as performance evaluation indexes. The index AP is the integral between 0 and 1 on the PR curve composed of Precision (P) and Recall ® of each category. Its calculation is shown in Formula (8). mAP is the mean of AP values of all categories, and its calculation is shown in Formula (9).

(8) $$\begin{array}{c} AP=\int_{0}^{1}P\left(R\right)dR \end{array}$$

(9) $$\begin{array}{c} mAP=\frac{\sum_{i=1}^{k}AP_{i}}{k} \end{array}$$

where Function P(R) is the *PR* curve with precision as the *y* axis and recall as the *x* axis, dR is the differential of *PR* curve on the *x* axis, $AP_{i}$ is the average precision of the *i*th target class, *k* is the total number of target categories.

#### 3.1.3. Model Training

The Mask R-CNN is trained on the dataset of express packages. The classification error, detection error, and segmentation error in the training process are recorded and the multi-task loss function *L* can be figured out, whose specific expression is shown in Formula (10).

(10) $$\begin{array}{c} L=L_{cls}+L_{box}+L_{mask} \end{array}$$

where $L_{cls}$ represents the classification loss function of Mask R-CNN, $L_{box}$ represents bounding box regression loss function, and $L_{mask}$ represents the mask regression loss function.

**Figure 5:**
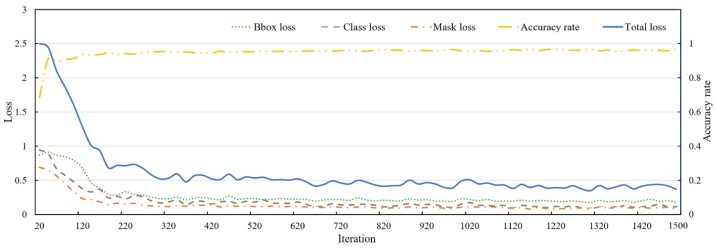
Variation of various loss and accuracy rates.

After 1500 iterations, the relevance of the three types of error values, the total error value and the accuracy rate value to the iteration process is shown in Figure 5. Where, the Bbox loss is the error value of $L_{box}$, the Class loss is the error value of $L_{cls}$, and the Mask loss is the error value of $L_{mask}$. As can be seen from Figure 4, the three kinds of errors tend to be stable when the iteration time is about 300, and the total errors tend to be stable when the iteration time is about 700. The convergence of training errors and accuracy rate are generally good, and the model has been well trained.

#### 3.1.4. Model Validating and Testing

The Mask R-CNN is trained, and then the object detection and instance segmentation accuracy of three types of packages with different iterations is obtained after model validating on validation dataset. When the iteration times are 300, 500, 700, 900, 1200 and 1500, the average precision of all kinds of targets on bounding box regression and instance segmentation are validated.

(1) Model validating

**Figure 6:**
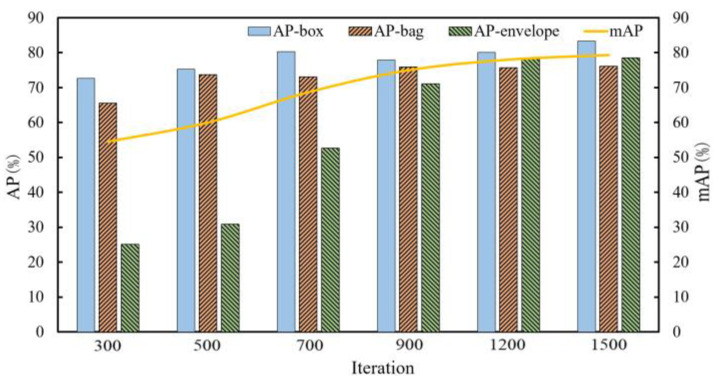
The bounding box regression average precision of various targets on the validation set.

**Figure 7:**
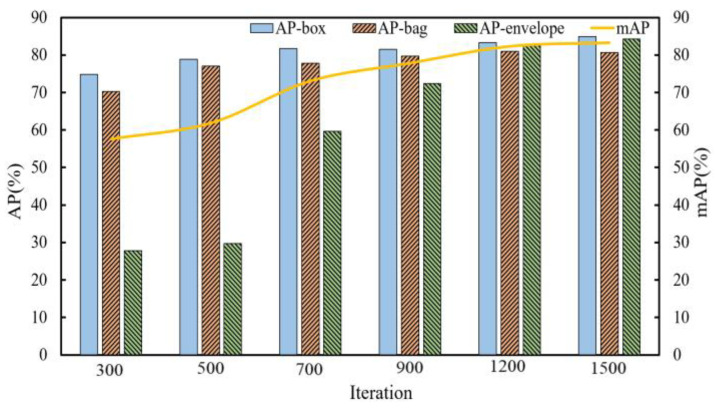
The instance segmentation average precision of various targets on the validation set.

For different target categories, IoU (Intersection over Union of the true bounding box over the predicted box) takes the experience value of 0.5 for validating and then statistical data has been recorded. The variation trend related to iterations based on the data is shown in Figure 6 and Figure 7.

As can be seen from Figure 6 and Figure 7, the AP and mAP of various targets in this dataset continuously improve with the increase in iterations, and the growth rate increases gradually before 900 iterations, and then slows down gradually between 900 and 1200 iterations. Finally, the precision values of various targets tend to stabilize around 1500 iterations.

(2) Comparative analysis of hyperparameter

In order to verify the influence of filtering positive and negative samples on the detection and instance segmentation of express packages, this research further changes the hyperparameter setting of the IoU threshold to conduct a comparison experiment. It is known that the smaller the IoU setting value is, the more negative samples will be filtered, and the experiment result is shown in Table 2.

It can be seen in Table 2, that filtering negative samples can improve the detection performance of the network to some extent. With the decrease in the IoU threshold, more false negative samples are filtered out, and the detection and segmentation performance of the model are gradually improved. When the IoU threshold is 0.1, the precision reaches the highest point, which makes the most obvious improvement on the detection and segmentation performance of the model. The average precision of various targets on the validation set is recorded when the IoU threshold is 0.1, as shown in Table 3. It can be seen from Table 3 that the AP and mAP of various targets are generally good, and the model is practicable in detection.

(3) Model testing

**Figure 8:**
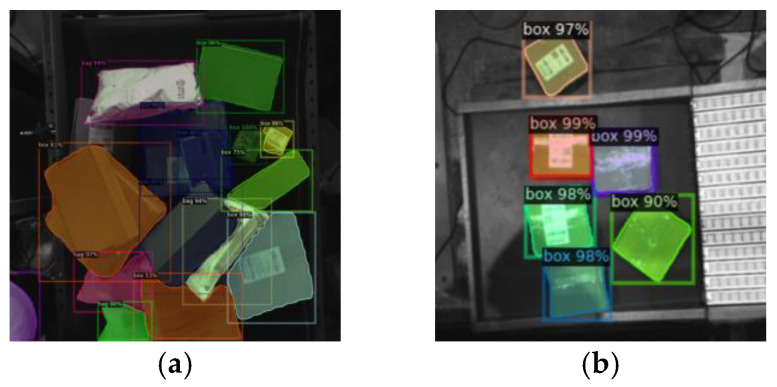

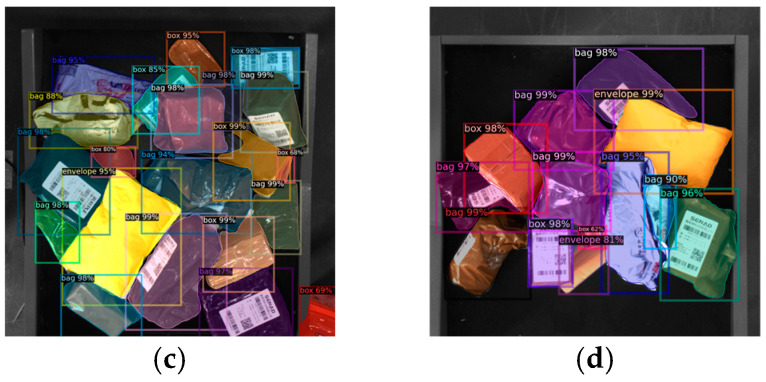
Detection effects of Mask R-CNN in testing. (**a**) Shadow. (**b**) Reflection. (**c**) Disordered stacking. (**d**) Overlapping and deformation.

The images of express packages under complex logistics sorting are used to test the Mask R-CNN. The detection effects in some typical complex scenes, such as shadow, reflection, disordered stacking, overlapping and deformation are obtained, as shown in Figure 8. It can be concluded from Figure 8 that Mask R-CNN has achieved good results in detection and instance segmentation of express packages in these complex scenes, and it is reliable and stable in MDFM for visual sorting.

#### 3.1.5. Performance Comparison with Classical Object Detection Algorithms

**Figure 9:**
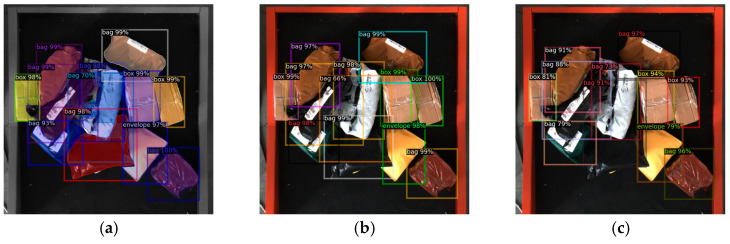
Schematic diagram comparing the detection effect with classic object detection algorithms. (**a**) Mask R-CNN. (**b**) Faster R-CNN. (**c**) RetinaNet.

In order to verify the superiority of Mask R-CNN in the detection and recognition ability of express packages compared with classical object detection algorithms in the actual complex logistics sorting scene, the same dataset is used for model training, validating and testing in this experiment. After 1500 iterations and the optimal hyperparameter settings being completed, respectively, different object detection algorithms, Mask R-CNN, Faster R-CNN, and RetinaNet, are experimented with under the same validation set. The results are shown in Table 4 and Figure 9.

It can be seen from Table 4 that Mask R-CNN has the best detection performance, whose mAP of the bounding box regression reaches 81.10%, increased by 0.93% and 1.01% compared with Faster R-CNN and the Retinanet, respectively. Also, it can be found from Figure 9 that Mask R-CNN achieves the best detection effect with more detected targets and higher recognition accuracy among the three object detection algorithms. In addition, Mask R-CNN has advantages in the instance segmentation module that divides the individual package units accurately, which makes it more suitable for actual complex logistics sorting scenes.

### 3.2. Robot Sorting Experiment

After detecting and recognizing the express package by Mask R-CNN, the sorting vector and optimal grasping position are determined in combination with the processed 3D point cloud data. On this basis, the robot sorting experiment is carried out to verify the effectiveness of the MDFM for visual sorting. In this experiment, a six-degree-of-freedom robot with a suction cup effector, an RGB-D camera and a sorting table were put into use, and the relevant experiment environment was built.

Express packages to be sorted in the experiment are boxes, bags and envelopes commonly used in actual logistics sorting, which are randomly distributed in different sizes, colors, shapes and stacked disorderly on the sorting table. The sorting experiment is divided into five groups, each group sorts 100 packages consecutively. In each group, the robot grasps a package one at a time, and the results have been calculated, as shown in Table 5. Another sorting experiment was conducted as a comparison using the method without point cloud data filtering, and the results are shown in Table 6. The sorting success rate compared with other methods is shown in Table 7.

According to Table 5, after five groups of sorting, the highest sorting success rate is 98% and the lowest is 96%, which shows that the MDFM for visual sorting achieves a success rate of 97.2% on average. Table 6 shows that the sorting success rate of the method without point cloud data filtering is 89.2%, which is 8.0 percentage points lower than that of MDFM. It can be inferred from Table 5 and Table 6 that point cloud data filtering contributes to obtaining more accurate sorting vector and optimal grasping position of express packages, and then improves the success rate of sorting in MDFM. Furthermore, Table 7 shows that the MDFM improves 2.9 and 7.5 percentage points, respectively, compared to the sorting methods proposed in [30,32]. The result indicates that the proposed method is more accurate in determining the optimal grasping position and the sorting vector.

In summary, based on the detection results of Mask R-CNN, reasonable sorting strategies for different kinds of express packages are adopted to determine the grasping order of packages in different states and positions, so as to complete the whole sorting with fewer detection times and faster sorting speed, and the sorting efficiency has been significantly improved. Therefore, the MDFM for visual sorting of express packages takes into account the high detection and recognition accuracy, high sorting success rate and high sorting efficiency of express packages, which is suitable for the automatic sorting of express packages during complex logistics sorting.

## 4. Conclusions

In this research, a new multi-dimensional fusion method for visual sorting of express packages under actual complex logistics sorting is proposed, in which Mask R-CNN is adopted and 3D point cloud data is used. Firstly, the express package images under the background of complex logistics sorting are collected, and the dataset is made. Secondly, Mask R-CNN is evaluated and applied to a 2D detection task. Then, the point cloud data is filtered, and a virtual grasping surface is fitted, after which accurate sorting information including the sorting vector and the optimal grasping position of express packages are worked out. Finally, robot sorting experiments are carried out. The main conclusions are as follows:

(1) The Mask R-CNN was evaluated for detection accuracy, achieving higher precision in object detection and having advantages in instance segmentation compared with previous classical object detection algorithms. The results show that Mask R-CNN can provide accurate detection information in MDFM.
(2) Based on accurate detection results, combined with precise vector sorting and optimal grasping position, the sorting success rate of the MDFM reaches 97.2%, proving the stability and applicability of the proposed sorting method.
(3) The method is conducive to improving the sorting efficiency of express packages under complex logistics sorting, and provides technical conditions for realizing comprehensive automation and high efficiency of sorting in complex scenes, which has important application value.

Although the MDFM proposed in this research improves the sorting efficiency of express packages, the actual logistics sorting scene will become more complex with the development of the logistics industry. Future work will further improve the method’s object detection precision of express packages and its adaptability in other complex logistics sorting scene datasets.

## Figures and Tables

**Table 1:** Number and proportion of various marked targets.

Target	Number	Proportion %
Box	3489	52.059
Bag	2741	40.892
Envelope	472	13.528
Total	6702	100.00

**Table 2:** Comparison experiment of different IoU threshold.

IoU Threshold	Bounding Box Regression mAP/%	Instance Segmentation mAP/%
0.9	73.67	77.50
0.7	78.09	81.96
0.5	79.34	83.29
0.3	80.13	84.01
0.1	81.10	85.10

**Table 3:** The average precision of various targets on the validation set (%).

	AP-Box	AP-Bag	AP-Envelope	mAP
Bounding Box Regression	83.769	77.301	82.233	81.10
Instance Segmentation	85.555	81.844	87.899	85.10

**Table 4:** Performance comparison with classic object detection algorithms.

Method	Type	Bounding Box Regression mAP/%	Instance Segmentation mAP/%	Weight Size/M	Average Single Inference Time /ms
Mask R-CNN	Two-stage	81.10	√	334.86	198
Faster R-CNN	Two-stage	80.17	-	314.83	205
RetinaNet	One-stage	80.08	-	288.98	182

The “√” in the table indicates that the module is available, and the “-” indicates that the module is unavailable.

**Table 5:** The results of sorting experiments based on MDFM.

Group	Results of Grasping		Sorting Success Rate (%)
	Success	Failure	
1	98	2	98
2	96	4	96
3	97	3	97
4	97	3	97
5	98	2	98
Total	486	14	97.2

**Table 6:** The results of sorting experiments without point cloud data filtering.

Group	Results of Grasping		Sorting Success Rate (%)
	Success	Failure	
1	90	10	90
2	91	9	91
3	89	11	89
4	89	11	89
5	87	13	87
Total	446	54	89.2

**Table 7:** Sorting success rate compared with other methods.

Method	Sorting Success Rate (%)
Method based on multi-modal information fusion [30]	94.3
Method based on multi-task deep learning [32]	89.7
Method without point cloud data filtering	89.2
Multi-Dimensional fusion method (Ours)	97.2

## Data Availability

The data used to support the findings of this study are available from the corresponding author upon request.
